# Dual-compressed photoacoustic single-pixel imaging

**DOI:** 10.1093/nsr/nwac058

**Published:** 2022-03-25

**Authors:** Yuning Guo, Baowen Li, Xiaobo Yin

**Affiliations:** Department of Mechanical Engineering, University of Colorado, Boulder, CO80309, USA; Department of Mechanical Engineering, University of Colorado, Boulder, CO80309, USA; Department of Material Science and Engineering, Department of Physics, Shenzhen Institute for Quantum Science and Engineering, Southern University of Science and Technology, Shenzhen518055, China; Department of Mechanical Engineering, University of Colorado, Boulder, CO80309, USA; Materials Science and Engineering Program, University of Colorado, Boulder, CO80309, USA

**Keywords:** photoacoustic imaging, compressive sensing, single-pixel imaging, surface tomography

## Abstract

Photoacoustic imaging, an acoustic imaging modality with potentially optical resolution in an optical turbid medium, has attracted great attention. However, the convergence of wavefront optimization and raster scanning in computational photoacoustic imaging leads to the challenge of fast mapping, especially for a spatial resolution approaching the acoustic deep-subwavelength regime. As a sparse sampling paradigm, compressive sensing has been applied in numerous fields to accelerate data acquisition without significant quality losses. In this work, we propose a dual-compressed approach for photoacoustic surface tomography that enables high-efficiency imaging with 3D spatial resolution unlimited by the acoustics in a turbid environment. The dual-compressed photoacoustic imaging with single-pixel detection, enabled by spatially optical modulation with synchronized temporally photoacoustic coding, allows decoding of the fine optical information from the modulated acoustic signal even when the variance of original photoacoustic signals is weak. We perform a proof-of-principle numerical demonstration of dual-compressed photoacoustic imaging, that resolves acoustic sub-acoustic-wavelength details with a significantly reduced number of measurements, revealing the potential for dynamic imaging. The dual-compressed concept, which transforms unobtrusive spatial difference into spatio-temporal detectable information, can be generalized to other imaging modalities to realize efficient, high-spatial-resolution imaging.

## Teaser

Dual-compressed photoacoustic imaging with single-pixel detection enables high-efficiency surface tomography with 3D spatial resolution unlimited by the acoustics, revealing the potential for dynamic imaging in a scattering medium.

## INTRODUCTION

Photoacoustic imaging (PAI) can image through an optically turbid medium and has found broad applications in biomedical diagnosis and remote sensing [1-3]. By exploiting spatio-temporal wavefront modulations of optical excitations, computational imaging techniques such as wavefront shaping [4,5] and speckle correlations [6,7] extract information from the scattered fields and have been recently explored for high-resolution PAI through turbid media [8-11]. In particular, taking advantage of the non-linear Grueneisen relaxation effect, photoacoustically guided wavefront shaping has achieved an acoustic resolution of ∼*λ_ac_*/4 [11]. However, computational PAIs benefiting from wavefront modulations converge gradually and, more severely, require time-consuming raster scanning. Here we propose a dual compressive sensing technique for fast 3D photoacoustic surface tomography and perform a proof-of-principle numerical demonstration of dual-compressed PAI with a resolution of <λ_ac_/10. In addition to the spatially optical wavefront modulations, the dual-compressed imaging uses a coded aperture to introduce randomized spatio-temporal delays to the locally generated photoacoustic signals. Upon mapping an image with sparsity, the correlations among the recorded photoacoustic signal, the optical random sampling and the acoustical spatio-temporal modulation allow the reconstruction of unobtrusive features through time-of-flight (TOF) measurement with a dramatically reduced number of measurements at a compression ratio as high as 21.9. The stochastically modulated time-varying acoustic signal amplifies the weak spatio-temporal variance and enhances image contrast. The dual-compressed photoacoustic imaging allows the optically encoded 3D, sub-acoustic-resolution information to be resolved acoustically with a single bucket detector, which is infeasible for the conventional compressed one even with a large number of iterations. The approach can also be generalized and broadly adapted to any imaging modalities that involve signal transductions by correlated spatio-temporal modulations of both excitations and signals.

Compressive sensing, a sparse sampling paradigm that accelerates data acquisition without a significant loss of quality [12], has facilitated the development of a variety of imaging techniques including magnetic resonance imaging [13], terahertz imaging [14], optical imaging [15], ultrasound imaging [16], etc. Using an array of ultrasound transceivers or patterned optical illuminations to construct measurement matrices, compressive sensing has been applied for rapid photoacoustic tomography [17-20], unfortunately with a limit in spatial resolutions. At high resolutions, small spatially optical differences and low optical absorption contrasts result in weak fluctuations in photoacoustically generated signals. It would require a vast number of measurements approaching the Nyquist limit or sometimes fail to converge a reliable image. The dual-compressed approach transforms the indistinctive optical variances into detectable temporal information in traveling low-frequency acoustic fields, differentiating the signals originating from different surface tomography features in the time domain and allowing high-quality image reconstruction with a substantially reduced number of measurements. Setting measurement matrices with patterned optical illumination and a coded acoustic aperture, the dual-compressed imaging allows high-efficiency detections with a single-pixel detector (a bucket detector [21-25]) and rapid 3D photoacoustic surface tomography with sub-acoustic-wavelength resolution.

## MODELING

**Figure 1.**
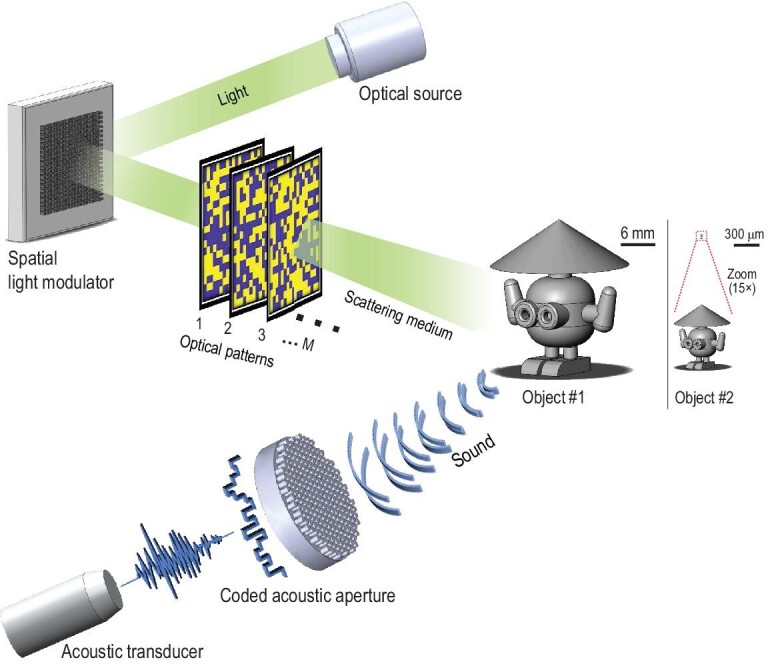
A schematic diagram of dual-compressed photoacoustic single-pixel imaging. A sequence of optical random patterns illuminate the object hidden behind/embedded in a scattering medium to generate acoustic waves. The generated time-varying photoacoustic signals are recorded by a single-element transducer after the modulation of a coded acoustic aperture.

In photoacoustic imaging, acoustic waves are generated from localized optical absorption via the thermo-elastic effect, enabling optical contrast identifiable in an acoustic way [26,27]. Figure 1 schematically depicts dual-compressed photoacoustic imaging using a single-pixel detector. A pulsed laser source with a high repetition rate is used for photoacoustic excitation. The light passing a spatial light modulator generates a sequence of random patterns to excite the photoacoustic signals from the surface of an object hidden behind/embedded in an optically scattering medium. The back-scattering ultrasonic waves are subsequently modulated by a coded acoustic aperture with tagging random time delays to the signals from spatial positions of the object. A large-aperture, single-element acoustic transducer is then used to collect spatiotemporally modulated photoacoustic signals. Herein, the optically patterned illumination implemented by the spatial light modulator serves as front-modulation in single-pixel imaging while the spatiotemporally acoustic modulation implemented by the coded acoustic aperture serves as back-modulation, forming dual compressive sensing in the proposed system.

According to the Poisson–Kirchhoff principle [28], for each measurement of compressed PAI with coded optical illumination, the transducer measures a time-varying photoacoustic signal, $u( t )$, derived from the sum of all waves starting with the same propagating distances, i.e. a linear superposition of photoacoustic waves ${C_{ij}}( t )$ originating from different spatial positions (*i, j*) in the tomographic image of a 3D object [29]. The acoustic TOF *t* carries the depth information of each spatial position. A coded acoustic aperture is then utilized to enhance the signal variance and increase image contrast. It stochastically introduces a uniformly distributed, spatial-dependent time delay ${T_{\mathit{ij}}}$ to the locally generated time-varying photoacoustic signal$\ {C_{\mathit{ij}}}( t )$, which yields the modulated signal ${\rm{as\ }}C_{\mathit{ij}}^{\prime}( {t^{\prime}} )$ and forms the recorded superposed signal $u^{\prime}( {t^{\prime}} )$. Here is the new TOF $t^{\prime} = t + {T_{\mathit{ij}}} = {g_{\mathit{ij}}}\ t$. For each measurement, ${g_{\mathit{ij}}}$ indicates a stochastically modulated spatial-dependent time delay spanning from original TOF *t* to ${g_{max}} \cdot t$ for a specific position (*i, j*). The amplification factor $g$ is defined as the statistical mean value of the dimensionless time delays, i.e. time delays $g\ = \langle {g_{\mathit{ij}}}\rangle \ = \langle 1 + {T_{\mathit{ij}}}/t\rangle \ \approx ( {{g_{max}} + 1} )/2$. It describes the modulation strength of the randomly coded acoustic aperture. Each sub-modulation region, i.e. the segment of the coded acoustic aperture corresponding to the spatial position (*i, j*), randomly adds a propagation path to the ultrasonic signal originating from that position and increases the TOF of the signal independently. As depth information can be extracted from the modulated TOF $t^{\prime}$, spatial information of a tomographic image V can therefore be obtained from the spatiotemporally resolved cross-correlation:

(1) \begin{eqnarray*}{{\rm{\hat{V}}}_{\mathit{ij}}}\left( {t^{\prime}} \right) &=& {\textit{corr}}\left[ {\Delta {\boldsymbol{S}},\Delta u^{\prime}\left( {t^{\prime}} \right)} \right]\nonumber\\ & =& \mathop \sum \limits_{m = 1}^M \Delta S_{ij}^m \cdot \Delta {u^{\prime m}}\left( {t^{\prime}} \right).\end{eqnarray*}

From a sequence of measurements *M*, where $\Delta \ S_{\mathit{ij}}^m = S_{\mathit{ij}}^m\ - \langle {S_{\mathit{ij}}}\rangle $ is the pattern-to-pattern variance between patterned illuminations and $\Delta {u^{\prime m}}( {t^{\prime}} )\ = {u^{\prime m}}\ ( {t^{\prime}} ) - \langle u^{\prime}( {t^{\prime}} )\rangle $ is the variance of the detected photoacoustic signal from the acoustic transducer.

The vector **v** containing N pixels represents the image V after basis sparse representation. The signal ***y*** after spatially optical sensing by the patterned illumination is ${\boldsymbol{y\ }} = \ {{\bf Sv}}$. The recorded signal **u** after temporally acoustic sensing by the coded aperture, which correlates with the sensed signal ***y***, can be depicted as ${{\bf u}}\ = {\rm{\ }}{{\bf T}}{\boldsymbol{y}} + {{\bf n}}\ = {\rm{\ }}{{\bf TSv}} + {{\bf n}}\ = {\rm{\ }}{{\bf Hv}} + {{\bf n}}$. The matrices **S** and ${{\bf T}}$ represent the transmission matrices produced by optical sensing and acoustic sensing, respectively. **H** is the effective measurement matrix that depicts the whole spatio-temporal sensing field in the 3D space and vector **n** denotes the noise. The measurement matrix **T** of the acoustic sensing, which bridges the tiny optical difference and identifiable acoustic information, needs to be calibrated in the experiment. The 2D ultrasonic field closely behind the coded acoustic aperture is spatially mapped as initial values by using a motorized translation stage and a hydrophone, then the fields of subsequent planes can be computed for populating the columns of measurement matrix **T** subsequently according to the angular spectrum algorithm [30]. For a sequence of measurements M, the signal **u** can be expressed as ${{\bf u}} = {[ {{{\bf u}}_0^{\rm{T}}{\rm{\ }}{{\bf u}}_1^{\rm{T}} \cdots {{\bf u}}_{{\rm{M}} - 1}^{\rm{T}}} ]^{\rm{T}}}\ $with a length of M × K, where K is the number of temporal sampling. The matrix ${{\bf H}}$ is constructed as ${{\bf H}} = {[ {{{\bf H}}_0^{\rm{T}}{\rm{\ }}{{\bf H}}_1^{\rm{T}} \cdots {{\bf H}}_{{\rm{M}} - 1}^{\rm{T}}} ]^{\rm{T}}}\ $with a size of (M × K) × N, where N is the number of image pixels. The compression ratio *γ*, representing the imaging efficiency of compressive sensing, is defined as the ratio of the image pixel counts N to the number of measurements M. Given the recorded signal **u** and the constructed matrix **H**, the sparse information ${{{\bf v}}^*}$ can be recovered by ℓ_1_-norm optimization v* = $\rm {arg} \mathop {min}\limits_{v}$||**v**||_ℓ_1__ subject to${\rm{\ }}{{\bf Hv}}\ = {\rm{\ }}{{\bf u}}$. The angular spectrum algorithm is utilized for the propagation of photoacoustic signals and the algorithm for signal reconstruction is based on the primal–dual algorithm utilized in the convex optimization solver [31].

Given the advantage of compressed single-pixel detection, the background noise induced by the scattering medium is weighted-sum by the bulk detector, which reduces its perturbation to the integrated photoacoustic signal in each measurement. As long as the statistical properties of the medium remain stationary and the number of speckle grains impinging on the active area of the object is high [32], the scattering medium introduces random noise to each measurement while it has limited influence on the cross-correlation of signal variance and pattern-to-pattern modulation among a sequence of measurements. As a result, the image can be reconstructed either in the case of the object embedded in the scattering medium (called the embedded case) or in the case of the object hidden behind the scattering medium (called the hidden case) by the proposed dual-compressed approach in a weak scattering medium. In our numerical demonstration, a random noise induced by the scattering medium and measurement is introduced as ambient noise to the signal, which applies to both the embedded case and the hidden case. The strength of the perturbation to generated photoacoustic signals is represented by a noise level of standard normal distribution that is randomly attached to the localized photoacoustic signals. Assuming the scattering medium is with a coherence length less than the size of the source segments on a spatial light modulator, the singular value distribution of the corresponding static transmission matrix follows a quarter-circle law [8]. The matrix elements are not significantly correlated and the scattering medium would not introduce spurious correlations for image reconstruction in this proof-of-principle demonstration of dual-compressed PAI systems.

## NUMERICAL RESULTS

**Figure 2.**
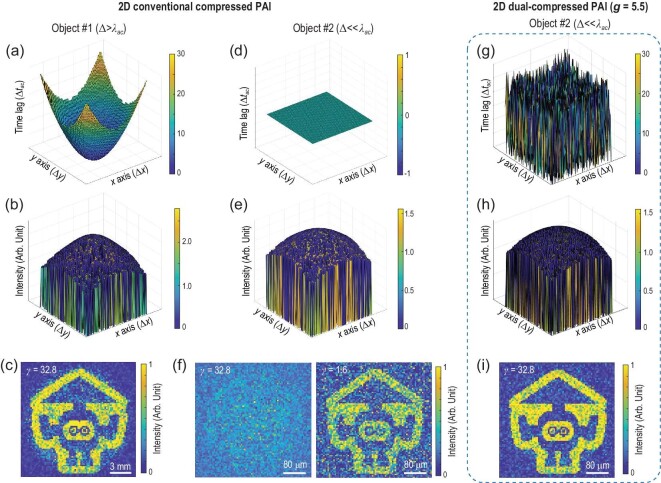
(a)–(f) 2D conventional compressed single-pixel PAI with only spatially coded optical illumination. (a) Time-lag distribution of photoacoustic signals, (b) intensity distribution of photoacoustic signals for a measurement and (c) the recovered image acquired from 2D imaging of Object #1 (pixel size ${\rm{\Delta \ }} = {\rm{\ }}200{\rm{\ \ \mu m}} > {\lambda _{ac}}$). The distribution of time lag and signal intensity possesses prominent gradients, allowing the recovery of the 2D image of Object #1 with a compression ratio of *γ* = 32.8. (d) Time-lag distribution of photoacoustic signals, (e) intensity distribution of photoacoustic signals for a measurement and (f) recovered images acquired from 2D imaging of Object #2 (${\rm{\Delta \ }} = {\rm{\ }}5{\rm{\ \ \mu m}} \ll {\lambda _{ac}}$) with *γ* = 32.8 and *γ* = 1.6, respectively. The time lag vanishes while the intensity distribution still possesses variances so that it demands a vast number of measurements to build the image of Object #2. (g)–(i) 2D dual-compressed single-pixel PAI with the amplification factor *g *= 5.5. (g) Time-lag distribution of photoacoustic signals, (h) intensity distribution of photoacoustic signals for a measurement and (i) recovered image acquired from 2D imaging of Object #2. The randomly added time delays enhance the signal variances, allowing the image of Object #2 with subwavelength details to be recovered with *γ* = 32.8.

When the acoustic modulation by the coded aperture is not performed, i.e. without introducing stochastically acoustic time delay, the dual-compressed PAI is degraded into the conventional compressed PAI that only employs spatially coded optical illumination or the configuration of the single-pixel camera [33-35]. Figure 2 theoretically compares the 2D imaging performance of the dual-compressed single-pixel PAI with the conventional compressed single-pixel PAI with spatially coded optical illumination. The conventional compressed PAI is first implemented on the 2D image of Object #1 with a pixel size Δ = 200 μm ($\Delta \ > {\lambda _{ac}}$, where ${\lambda _{ac}}$= 148 μm is the wavelength of the acoustic detection in water) and Object #2 with a pixel size Δ = 5 μm ($\Delta \ll {\lambda _{ac}}$), as shown in Fig. 1. For 2D imaging of Object #1 (Fig. 2a–c), the backscattered photoacoustic signals at the observation plane (a plane perpendicular to the propagation axis and before the detector) in a measurement displays prominent time lags Δ*t* with the unit of acoustic period $\Delta \ {t_{ac}} = \ 1 / {f_{ac}}$ where ${f_{ac}} = \ 10{\rm{\ MHz}}$ is the detection frequency. The acoustic intensity of the photoacoustic signal also possesses obvious variances within the field of view. It is sufficient to reconstruct the 2D image with a high compression ratio *γ* = 32.8. However, for 2D imaging of Object #2 (Fig. 2d–f), time lags Δ*t* vanish due to the indistinguishable time difference of acoustic TOFs from different spatial positions. Since the TOF of a photoacoustic signal *t* is in direct proportion to the acoustic propagation distance *d*($t \propto d$), the time lag of traveling signals from neighboring spatial positions is <3.4 ns, which is far beyond the acoustic resolving capability. Nevertheless, the intensity distribution still demonstrates variances because the intensity of the ultrasonic wave *I* is more sensitive to propagation distance *d* according to the approximation of the inverse-square law ($I \propto 1/{d^2}$). Thus, the 2D image with subwavelength details could be built with a low efficiency by abundantly cumulating the weak signal fluctuations. As Fig. 2f shows, it is hard to extract effective information from the image built with the same high compression ratio *γ* = 32.8. With a greatly increased number of measurements, the image can be recovered with a compression ratio *γ* = 1.6 as well as a peak signal-to-noise ratio (SNR) 3 dB lower than the recovered image of Object #1. However, upon such a low compression ratio, the advantage of compressed sensing is lost in this subwavelength compressed PAI. Although the conventional compressed single-pixel PAI can capture subwavelength details owing to the sub-acoustic-resolution information being encoded by fine optical illumination patterns that can be resolved from photoacoustic signals with weak fluctuations via correlation analysis, it compromises with a vast number of measurements and loses its major advantages of efficient data acquisition.

By locally introducing randomized temporal tagging on traveling acoustic waves, a coded acoustic aperture with an amplification factor *g *= 5.5 is utilized in dual-compressed single-pixel PAI for 2D imaging of Object #2 (Fig. 2g–i). Assuming the same energy loss of the signals during the acoustic temporal modulation, the variance of signals intensity would change little. The stochastically added local time delays greatly increase the time differences of acoustic TOFs from spatial positions that form a randomly distributed time-lag field with strong variances as shown in Fig. 2g. Thus, with enhanced signal variances, the dual-compressed approach allows the 2D image of Object #2 with subwavelength details to be recovered with the spatial compression ratio *γ* = 32.8, i.e. only 5% of measurements are required in comparison to the conventional compressed approach with only coded optical illumination, which greatly improves the imaging speed.

When a high spatial resolution is desired or the object has a low optical absorption contrast, the signal variance of the conventional compressed PAI could be weak, leading to low imaging speed. The proposed dual-compressed PAI is aimed to regain the advantage of efficient data acquisition and efficiently achieve 2D sub-acoustic-resolution imaging based on the enhanced correlation of measurements even for the conditions of low signal variances. Furthermore, for the conventional compressed PAI, the photoacoustic signals generated from acoustic-subwavelength-sized details would also result in an indistinguishable time difference of acoustic TOFs, which severely hinders its depth-resolved ability and thus limits its capability of 3D PAI imaging. In contrast, the proposed dual-compressed approach provides tunable acoustic TOFs so that the time lags can be resolved, revealing the feasibility of 3D subwavelength PAI.

**Figure 3.**
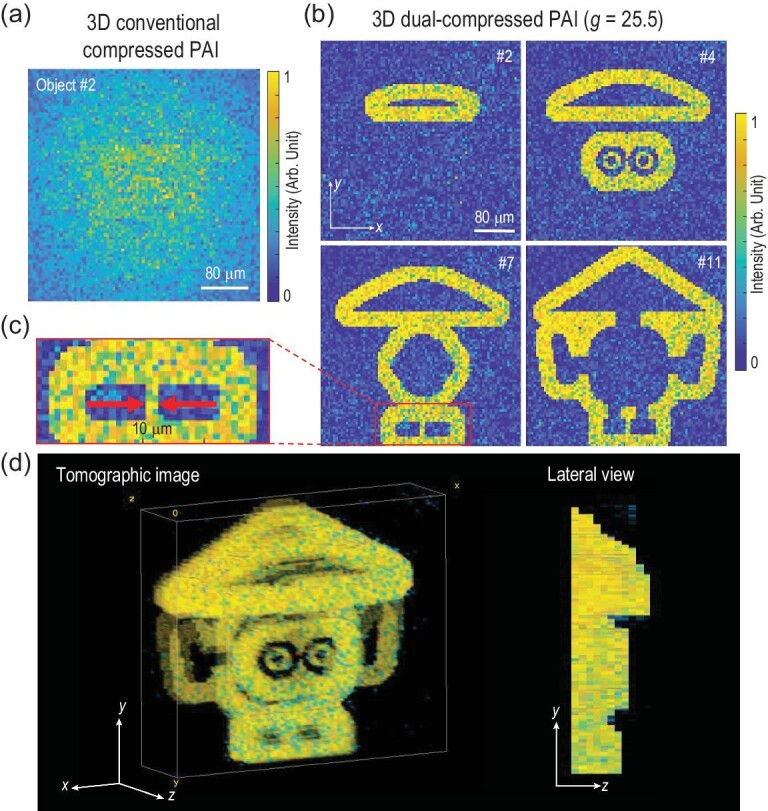
(a) 3D imaging of Object #2 based on conventional compressed PAI. The depth information is indistinguishable, which leads to tomographic images overlapping, releasing no effective information. (b)–(d) 3D imaging of Object #2 based on dual-compressed single-pixel PAI with *g* = 25.5. (b) Four recovered image slices with depth locations of 10, 30, 60 and 100 μm (slices numbers #2, #4, #7 and #11) far from the forefront of the object, respectively. The information of each image slice can be identified, which indicates an axial resolution (along the depth direction) of 10 μm. (c) Zoomed-in recovered image slice shows the detail with a width of 10 μm, which indicates the 3D spatial resolution reaching the subwavelength range. (d) The reconstructed tomographic image and its projection on the *yz* plane, which indicates that a greatly reduced number of measurements with a compression ratio of *γ* = 21.9 is enough to build a surface tomographic image with 3D spatial resolution unlimited by acoustics.

For 3D tomographic imaging based on conventional compressed single-pixel PAI with only spatially coded optical illumination, since the subwavelength details of the cross-sectional image slices of Object #2 are indistinguishable along the axial direction for 10 MHz acoustic detection, the images at different depths are overlapped and no effective information is released, as Fig. 3a shows. To address the issue of 3D subwavelength PAI, a coded acoustic aperture with strong modulation of *g *= 25.5 is employed in dual-compressed PAI to demonstrate 3D imaging capability upon the same number of measurements in 3D conventional compressed PAI. Eleven cross-sectional image slices are selected from Object #2 with a depth interval of 10 μm. Each measurement collects the coded information from the 3D illuminated positions including the pixels at different depths and the surface topographic imaging of a 3D object is then built using the measurement correlation. As a demonstration, four recovered slices with depth locations of 10, 30, 60 and 100 μm far from the forefront of the object, respectively, are displayed in Fig. 3b. The identifiable information from each image slice indicates an axial resolution (along with depth in the *z* direction) of 10 μm. The zoomed-in image slice in Fig. 3c also shows the detail with a width of 10 μm which indicates that 3D spatial resolutions including the transverse resolution and axial resolution surpass the acoustic resolution limit via dual synchronously compressed operations. The morphological characteristics of the reconstructed tomographic Object #2, composed of 11 image slices, can be recognized clearly in Fig. 3d. For 3D surface tomography, the optical pattern can only illuminate the front side of the object with a convex geometry, which means each lateral position on the *xy* plane corresponds to a unique depth in the *z* direction. The image pixel counts in surface tomography are the pixel number of one slice instead of the total pixel counts from all slices in a measurement. Thus, the 3D tomographic images are recovered with a compression ratio of *γ* = 21.9 here, which demonstrates that 3D surface tomographic subwavelength PAI can be realized by a small number of measurements with a high compression ratio. By increasing the time lag of TOFs from spatial positions with different depths, the coded acoustic aperture allows subwavelength depth information to be distinguished, which increases signal variance spatiotemporally and strengthens the image contrast of the tomographic slices. Thereby, the proposed dual-compressed single-pixel PAI, which transforms the 3D indistinctive difference of optical responses into spatio-temporal detectable acoustic information, enables 3D subwavelength surface tomographic PAI with dramatically accelerated data acquisition.

**Figure 4.**
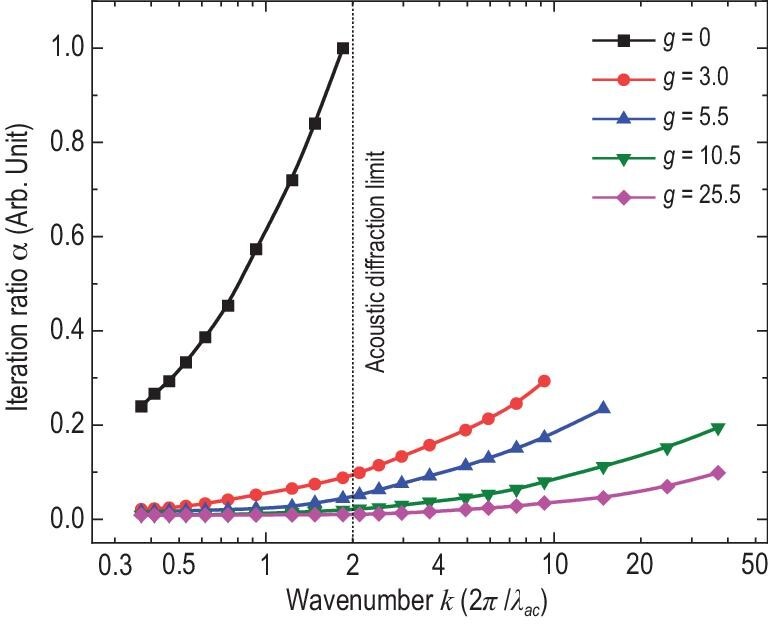
3D imaging capability comparison of compressed PAI systems. The coded acoustic aperture with amplification factors *g =* 0, 3.0, 5.5, 10.5 and 25.5, respectively, is utilized to provide different modulation strengths. The imaging resolution *R* is converted into the wave number as $k = ( {{\lambda _{ac}}/R} ) \cdot {k_{ac}}\ $in the abscissa axis for convenience. The black dashed line denotes the acoustic diffraction limit ${\lambda _{ac}}/2$, which corresponds to a wave number $k$ = 2${k_{ac}}$. For conventional compressed PAI (*g *= 0 case), the number of required measurements for image reconstruction increases rapidly with the wave number and the imaging becomes invalid when the threshold of acoustic diffraction limit is approaching. For dual-compressed PAI (*g *= 3.0, 5.5, 10.5 and 25.5 cases), the required measurements are kept at a small value with an iteration ratio of 0.01∼0.10 before the threshold and it still works well for large wave-number conditions, i.e. finer spatial resolutions.

Figure 4 compares the 3D imaging efficiency of compressed PAI systems. The coded acoustic aperture with the amplification factors *g* = 0, 3.0, 5.5, 10.5 and 25.5, respectively, is utilized to provide different modulation strengths. The spatial resolution *R* of the imaging is converted into the wave number as $k = \frac{{2\pi }}{R} = \frac{{{\lambda _{ac}}}}{R}{\rm{\ }} \times \frac{{2\pi }}{{{\lambda _{ac}}}} = \frac{{{\lambda _{ac}}}}{R} \times {k_{ac}}$ in the abscissa axis for convenience, which means that a larger wave number corresponds to a better spatial resolution. The iteration ratio $\alpha $ is defined as $\alpha = {M_i}/{M_{max}}$ for convenient comparison of the number of required measurements, where ${M_i}$ is the required number of measurements of the *i*-th test and *M_max_* is the maximum number of measurements among the tests. Instead of calculating a full-field angular spectrum algorithm, the propagation of ultrasonic waves following the inverse-square law and a noise level of 0.1 are utilized on the transmission of photoacoustic signals to reduce the simulation cost when the number of measurements is large.

For *g* = 0, i.e. the conventional compressed single-pixel PAI without TOF modulation, the required iteration ratio for successful reconstruction of a 3D tomographic image increases rapidly with the increasing wave number approaching a finer resolution. The conventional approach becomes invalid when the wave number approaches the threshold $k\ = \ 2{k_{ac}}$, corresponding to the acoustic resolution limit ${\lambda _{ac}}/2$, whereas for the proposed dual-compressed single-pixel PAI, the required number of measurements remains small with the iteration ratio *α* ranging from 0.01 to 0.1 when the wave number is smaller than the threshold, which greatly speeds up data acquisition in 3D imaging. Dual-compressed PAI still works well for the sub-acoustic-resolution condition corresponding to a wave vector $k > 2{k_{ac}}$, which makes 3D subwavelength PAI achievable with a slowly increased number of measurements below an iteration ratio of 0.3. For the dual-compressed PAI, a larger *g* indicates a stronger temporal modulation so that a low iteration ratio is required and a better resolution can be achieved efficiently. Taking the *g *= 5.5 case as an example, the realizable resolution is improved 10 times and <1/4 measurements are required to construct a tomographic image in comparison with the *g* = 0 case. Thus, the dual-compressed approach paves a way for high-efficiency surface tomographic imaging in which the resolution problem of 3D subwavelength PAI is transferred away from the theoretical limit towards the manufacturing level of acoustic modulation.

## DISCUSSION

The acquisition time of each measurement is the duration of the photoacoustic signal from generation to detection, which is related to the temporal length of the signal, the propagation distance, as well as the time delay introduced by the coded acoustic aperture. Besides the acquisition time for each measurement, the total acquisition time of the imaging process also depends on the refresh rate of the spatial light modulator and coded acoustic aperture, as well as the required number of measurements. In our proposed dual-compressed PAI system, the ultrasonic temporal measurement is a few milliseconds for a propagation distance of tens of centimeters in water even incorporating the additional propagation time generated by the coded acoustic aperture for a single measurement. Assuming the frame rate of the dual-compressed PAI is not limited by the refresh rate of the spatial light modulator and the designed coded acoustic aperture, the imaging speed of ∼300 frame/s would be achieved theoretically, revealing the potential of fast imaging.

The dynamic scattering medium changes the distribution of the optical fluctuation in a scattering medium on the millisecond timescale, while the global noise level would be stable owing to a spatial intensity integration is performed by the non-pixelated detector. To satisfy the requirements of real-time imaging, multiple approaches can be employed to potentially improve the imaging efficiency including optimizing the optical sampling basis, utilizing the spatial light modulator or any equivalent component with a higher refresh rate, seeking a tradeoff between the modulation strength and the introduced time delays in the coded acoustic aperture, etc. In particular, we believe that optimized deterministic basis patterns, e.g. Fourier basis, Hadamard basis and Haar wavelet basis [23,36,37], have the capability to improve the imaging speed in basis-projection single-pixel imaging. Since the goal of our work is to provide a prototype of dual-compressed single-pixel PAI instead of optimizing its imaging efficiency, a universal basis–random pattern basis is used as the input basis for the general demonstration due to its weak coherence with the sparsity constraint of the signal in the transformed domain [38]. Future work may consider careful optimization of the basis for obtaining higher efficiency or better resolution.

Here, we provide some information regarding the selection of key components in the potential dual-compressed single-pixel PAI experiment. To start with, the selection of the optical excitation wavelength matters in the experiment since it would tune the spectrum-dependent absorption of the chromophores and affect the light-penetration depth as well as the point spread function. Then, a spatial light modulator (or digital micromirror device) with megapixels, where the pixels are divided into the desired individual segments for reducing the controllable degree of freedom, can be used for coding a set of microstructured projecting patterns sequentially. Next, the presence of a scattering medium around the object, even in the dynamical scattering case, does not invalidate the operation principle of the proposed dual-compressed scheme with single-pixel detection, yet strong scattering may lead to complete reconstruction failure. The compressive algorithm can suppress random noise in the reconstruction process, but the denoising process still needs to be considered for other kinds of noise suppression in the compressed single-pixel imaging experiment [39,40]. Furthermore, multiple approaches can be implemented to decrease the possible coherence of the voxels in the coded acoustic aperture by the approaches such as randomly distributing the voxels in the lateral plane, selecting different parts of the aperture for each modulation or introducing rotation as an additional degree of freedom. The coded acoustic aperture can be packaged and mounted on a 3D motorized translational stage with multi-axis configurations, which allows the synchronous control of the refresh of both the optical random pattern and acoustic modulation for each measurement. The field of view of the dual-compressed imaging would be determined by the smaller value of the array size of the spatial light modulation and the aperture size of the coded acoustic aperture. In addition, either the focused transducer with improved detection sensitivity or the unfocused one specialized for more sound energy collection works in the experiment as a single-element transducer. The selection of the transducer depends on specific conditions including the strength of the photoacoustic signal and the acoustic impedance matching.

The dual-compressed PAI with a bucket detector brings in a promising approach to realize efficient 3D tomographic imaging with a spatial resolution much lower than the acoustic diffraction limit. However, several issues that may hinder the practical implementation of compressed PAI need careful consideration. First, for a deep-subwavelength acoustic resolution, elaborate optical illumination in which the segment size may approach the level of the optical wavelength is desired, which could make it difficult to generate localized distinguishable photoacoustic signals from different positions of the object. Second, to guarantee apparent signal variances as well as enhance the resolved capability of low-frequency detection, a powerful ultrasonic modulation provided by a coded acoustic aperture with an extremely fine structure and good guiding capability of the acoustic wave is desired, which poses a challenge for the practical design and fabrication. Techniques such as the acoustic delay line [41,42], phase non-uniformity microstructure [16], coiling-space or transformation-based metamaterial [43,44], acoustic resonators [45,46] and ergodic relay [47] may provide references for the practical design of a coded acoustic aperture. Besides, since the center frequency of ultrasonic waves varies with the absorber's size in the photoacoustic generation process [48], the matched bandwidth of the transducer and the photoacoustic signal is expected to optimize the signal-to-noise ratio and thus increase detection sensitivity [49]. Furthermore, the size of the measurement matrix increases with the number of measurements in the dual-compressed PAI. To resolve a complex object with fine details, a large measurement matrix would increase the computation cost greatly and prolong the image reconstruction time. Advanced numerical algorithms need to be exploited for applications in real-time environments. Under the tunable amplification capability of signal fluctuations, the dual-compressed concept can be generalized to universal imaging techniques, especially those with signal transductions, by correlated samplings for exceptional imaging performance.

## CONCLUSION

In conclusion, we report a theoretical dual-compressed framework on photoacoustic imaging that realizes high-efficiency 3D subwavelength surface tomographic imaging. Besides spatially patterned illumination, dual-compressed single-pixel PAI employs a coded acoustic aperture to stochastically introduce local time delays, allowing the TOFs of spatial-dependent photoacoustic signals to be distinguished temporally. The enhanced signal variance along with measurement correlation facilitates image reconstruction in the compressed system with accelerated data acquisition. The dual-compressed approach allows the optically encoded sub-acoustic-resolution information to be resolved acoustically via strongly enhanced correlation analysis, which enables 3D subwavelength surface tomographic imaging with a resolution of <λ_ac_/10 upon a dramatically reduced number of measurements with a compression ratio as high as 21.9. The dual-compressed approach can be generalized to other imaging techniques involved in signal transductions for exceptional performance.
